# Isolated Follicles Enriched for Centroblasts and Lacking t(14;18)/BCL2 in Lymphoid Tissue: Diagnostic and Clinical Implications

**DOI:** 10.1371/journal.pone.0151735

**Published:** 2016-03-18

**Authors:** Grant E. Nybakken, Rajeev Bala, Dita Gratzinger, Carol D. Jones, James L. Zehnder, Charles D. Bangs, Athena Cherry, Roger A. Warnke, Yasodha Natkunam

**Affiliations:** Department of Pathology, Stanford University School of Medicine, Stanford, California, 94305, United States of America; The University of North Carolina at Chapel Hill, UNITED STATES

## Abstract

We sought to address the significance of isolated follicles that exhibit atypical morphologic features that may be mistaken for lymphoma in a background of reactive lymphoid tissue. Seven cases that demonstrated centroblast-predominant isolated follicles and absent BCL2 staining in otherwise-normal lymph nodes were studied. Four of seven cases showed clonal B-cell proliferations amid a polyclonal B cell background; all cases lacked the IGH-BCL2 translocation and BCL2 protein expression. Although three patients had invasive breast carcinoma at other sites, none were associated with systemic lymphoma up to 44 months after diagnosis. The immunoarchitectural features of these highly unusual cases raise the question of whether a predominance of centroblasts and/or absence of BCL2 expression could represent a precursor lesion or atypical reactive phenomenon. Differentiating such cases from follicular lymphoma or another mimic is critical, lest patients with indolent proliferations be exposed to unnecessarily aggressive treatment.

## Introduction

Unusual growth patterns of atypical large cells associated with lymph node follicles have been previously described and range from reactive hyperplasias to germinotropic lymphomas. In recent years, recognition of aberrant strong coexpression of the germinal center marker CD10 and the anti-apoptotic protein BCL2, has been described within isolated secondary follicles of otherwise-normal lymph nodes.[1] Since its recognition, this phenomenon, variously termed ‘*in situ* localization of follicular lymphoma’, ‘follicular lymphoma *in situ*’ (FLIS) or ‘follicular lymphoma-like lesion of uncertain clinical significance’, has been described in histologically unremarkable lymph nodes from healthy patients, normal lymph nodes in patients with a prior or concurrent diagnosis of follicular lymphoma (FL), lymph nodes involved by a variety of other hematolymphoid malignancies, and lymph nodes removed during the evaluation of non-hematolymphoid neoplasms.[1–9]

The clinical significance of FLIS is still of uncertain significance, particularly because numerous studies have reported the presence of non-neoplastic cells that harbor *IGH-BCL2* gene rearrangements in the peripheral blood and tissues of patients with no evidence of FL.[10–20] Diagnostic criteria for identifying FLIS and guidelines for distinguishing this pattern of involvement from cases of partial lymph node involvement by FL have been proposed in an attempt to stratify patients into risk groups.[21–23] To date the reported cases in the literature contain aberrant strong coexpression of CD10 and BCL2 within follicles containing predominantly centrocytes amid otherwise-normal lymph nodes.[3–9, 21]

Herein, we report seven cases characterized by isolated abnormal follicles composed of atypical centroblasts within otherwise-normal lymph nodes that show significant differences from previously described cases. Detailed morphologic and immunophenotypic characterization was undertaken, and in cases with sufficient tissue, cytogenetics and molecular studies for clonal *IGH@* gene rearrangements were performed. The diagnostic challenges and clinical implications of these unusual findings are described.

## Materials and Methods

### Case Selection

A search of the Department of Pathology database at Stanford University Medical Center to include the terms “atypical follicles”, "follicular lymphoma in situ," and "follicular lymphoma-like B-cells", yielded cases received between 2010 and 2014. These showed either an abnormal pattern of strong CD10 and BCL2 coexpression within otherwise-normal secondary follicles or isolated abnormal follicles composed predominantly of centroblasts within lymph nodes with preserved architecture. The latter group of cases was selected for inclusion. Institutional Review Board approval was obtained from Stanford University for these studies. Clinical information including follow-up in the form of subsequent lymph node samples, staging bone marrow biopsies, imaging studies and clinical evaluations was recorded. Patient records and information were anonymized and de-identified prior to analysis.

### Histologic Assessment

The morphologic characteristics of each case were recorded in a manner similar to reports of follicular lymphoma in situ,[21] including the total number of follicles within the lymph node, the number of abnormal follicles involved, and the grade (ie: number of centrocytes and centroblasts) of the abnormal follicles, as defined by the 2008 World Health Organization classification.[24]

### Immunohistochemistry

Immunohistochemical stains were performed on 4-micron thick, formalin-fixed, paraffin-embedded whole tissue sections using automated staining platforms (BenchMark XT, Roche/Ventana Medical Systems, Tucson, AZ or Leica BOND-MAX, Leica Microsystems Inc, Buffalo Grove, IL). Primary antibodies and conditions used for immunohistochemistry are detailed in Table 1 and were performed in all cases where involved follicles were present on sufficient numbers of sections. Interpretation of staining intensity and patterns for CD10, BCL2, IgM, HGAL and Ki67 in lesional follicles was performed by comparison to background reactive secondary follicles, particularly, mantle zone B-cells and T-cells, respectively.

**Table 1:** Reagents and Conditions Used for Immunohistochemistry.

Antibody	Clone/Source	Dilution	Platform and Pretreatment
**CD20**	L26^a^	1:1000	BOND-MAX, ER2 retrieval
**CD3**	Rabbit Polyclonal^b^	1:100	BenchMark XT, Standard retrieval
**CD10**	56C6^c^	1:20	BOND-MAX, ER2 Retrieval
**BCL2**	124^a^	1:50	BenchMark XT, Standard retrieval
**BCL6**	GL191E/A8^b^	1:100	BenchMark XT; Standard retrieval
**HGAL**	MRQ-49^b^	1:100	BOND-MAX, ER2 retrieval
**IgM**	Polyclonal^a^	1:1250	BenchMark XT; Standard retrieval
**IRF4**	MUM1P^a^	1:40	BOND-MAX, ER1 retrieval
**Ki67**	MIB-1^a^	1:200	BOND-MAX, ER2 retrieval
**CD21**	IF8^a^	1:80	BOND-MAX, ER2 retrieval
**CD23**	1B12^c^	1:50	BOND-MAX, ER2 retrieval
**D2-40**	D2-40^a^	1:80	BOND-MAX, no retrieval
**Kappa**	Rabbit polyclonal^a^	1:1000	BenchMark XT; Protease 2 retrieval
**Lambda**	Rabbit polyclonal^a^	1:4000	BenchMark XT; Protease 2 retrieval

^a^Dako, Carpinteria, CA

^b^Cell Marque, Rocklin, CA

^c^Novocastra, Newcastle upon Tyne, UK

### Fluorescence *In situ* Hybridization (FISH)

Four micron formalin-fixed, paraffin-embedded tissue sections were analyzed for *BCL2*, *IRF4* and *BCL6* gene rearrangements by fluorescence *in situ* hybridization using a 5’/3’ breakapart probes for the *BCL2* gene (ZytoVision, Bremerhaven, Germany), *IRF4* gene (Empire Genomics, Buffalo, NY) and the *BCL6* gene (ZytoVision). Briefly, using a Vysis VP2000™ slide pretreatment instrument and reagents (Abbott Molecular, Chicago, IL), slides were deparaffinized with CitroSolv™, digested with a 10% pepsin solution at 37°C, pre-treated with a sodium thiocyanate solution at 80°C, re-fixed in 10% buffered formalin, and dehydrated in an ethanol series. Dried, dehydrated slides were mounted with probe solution per manufacturer’s instructions, denatured with a Vysis^®^ HYBrite instrument at 80°C for six minutes and hybridized for 48 hours at 37°C. Slides were washed with 2xSSC/0.3% NP-40 at 73°C for two minutes, counterstained with DAPI and analyzed with an Olympus BX51 microscope equipped with an 100x oil immersion objective, appropriate fluorescence filters and CytoVision^®^ imaging software (LeicaBiosystems, Buffalo Grove, IL).

### Genotyping

Polymerase chain reaction (PCR) for clonal *IGH@* gene rearrangements was performed on DNA obtained from paraffin-embedded tissue samples in a subset of cases using the IGH Gene Rearrangement Assay (InvivoScribe, San Diego, CA) according to the manufacturer’s instructions. Three cases (cases #2, 3 and 5) in which unstained sections were available were further evaluated by microdissection. To accomplish this, unstained sections were overlaid on H&E stained sections and the area of interest marked and removed by scraping. Desired portions were extracted using proteinase K digestion followed by QIAgen DNA column purification (QIAgen Inc, Valencia, CA). Elution volume of 22uL with heat and shaking were used with a yield of 19uL. Clonality was assessed using Invivoscribe B-clonality kit, primer mix A (FR1-JH) and primer mix F (amplification control). Tube F was used to assess amplifiability and to estimate yield. No spectrophotometric assessment of yield was performed due to very low amount of sample available for these assays. Uninvolved nodes were subjected to clonality assessment in parallel. All except the uninvolved sample of case 5, among the three paired samples yielded amplifiable DNA. Analyses of amplicons were performed by fragment analysis using capillary electrophoresis on the ABI 3100 Genetic Analyzer (Applied Biosystems, Foster City, CA).

## Results

### Clinical Features

The seven patients in this cohort included five women aged from 51 to 61 years old and two men aged six and 61. Anatomic sites of involvement included peripheral as well as internal sites such as periaortic lymph node and rectal mucosa-associated lymphoid tissue. Six cases were sampled during evaluation for unrelated medical conditions (three axillary lymph node dissections for invasive ductal carcinoma of the breast, one periaortic lymph node in a patient with cholecystitis, a tonsillectomy in a six-year old boy, and a colonoscopic biopsy for workup of intractable diarrhea) and seventh was a cervical lymph node removed for lymphadenopathy. Although three of the patients had invasive ductal carcinoma of the breast, none of the patients had associated follicular lymphoma (or other subtypes of lymphoma), detected either in the same lymph node or systemically at the time of biopsy. Bulky lymphadenopathy was not detected in any of the patients in our cohort. A summary of clinical features and follow-up is presented in Table 2.

**Table 2:** Summary of Clinical and Histologic Findings.

Case	Age/Sex	Anatomic site	Degree of Involvement by atypical follicles	Clinical Information and Follow-Up
**1**	61 F	Axillary lymph node	<5 follicles; <50%	Remote history of radiation for cutaneous T-cell lymphoma; breast carcinoma; no cutaneous or systemic lymphoma at diagnosis or at 44 months.
**2**	53 F	Axillary lymph node	<5 follicles; <50%	Breast carcinoma; no systemic lymphoma at diagnosis or at 14 months.
**3**	58 F	Axillary lymph node	<5 follicles; <50%	Breast carcinoma; no systemic lymphoma detected at diagnosis
**4**	53 F	Periaortic lymph node	<5 follicles; <50%	No systemic lymphoma detected at diagnosis
**5**	6 M	Tonsil	<5 follicles; <50%	No systemic lymphoma detected at diagnosis or at 27 months.
**6**	51 F	Rectum	<5 follicles; >50%	No systemic lymphoma detected at diagnosis
**7**	61 M	Cervical lymph node	<5 follicles; <50%	No systemic lymphoma detected at diagnosis or at 2 months.

### Histologic Features

**Fig 1:**
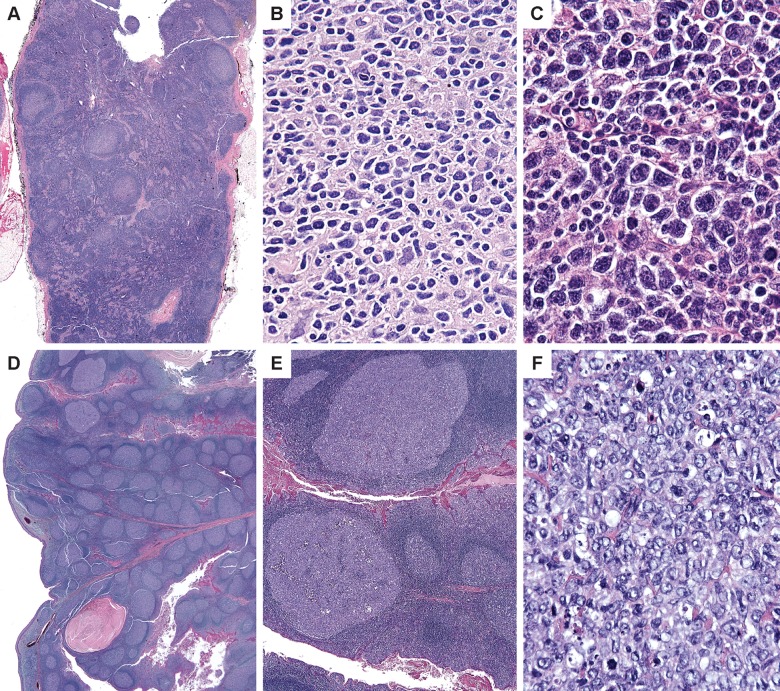
Morphology of isolated atypical follicles. An axillary lymph node dissection in a 61 year-old woman with breast carcinoma (case 1) shows one lymph node with scattered follicles containing sheets of centroblasts (A). The involved follicles exhibit sheets of large atypical cells with highly pleomorphic nuclear outlines and atypical mitoses (B). An axillary lymph node from a 53-year old woman (case 2) shows highly atypical large cells occupying an involved follicle (C). Sections of tonsil in a 6-year old boy (case 5) demonstrate a background of reactive follicular hyperplasia within which isolated follicles (upper left) show sheets of centroblasts (E and F).

The seven cases in our series showed scattered follicles composed almost exclusively of centroblasts localized to isolated follicles in lymph nodes with preserved normal architecture (Fig 1). Morphologically, the cases demonstrate abnormal follicles composed of a cluster of cytologically atypical centroblasts. In each case, these follicles were few and comprised a minority of the node (<5%) with surrounding germinal centers demonstrating normal cytologic and architectural features. Although a diagnosis of partial involvement by follicular lymphoma was considered, these follicles were scattered singly throughout the nodes, a pattern that is not in keeping with that consideration. These follicles, however, lacked normal components of germinal centers with no tingible body macrophages and absent polarization (Fig 1).

Cytologically, the centroblasts were enlarged with vesicular chromatin and prominent nucleoli. They demonstrated nuclear membrane irregularity and deviated from the morphology seen in the surrounding normal lymph node. Furthermore, atypical mitoses were appreciated with admixed tingible body macrophages but lacking centrocytes. Overall, these features, in the context of follicular lymphoma, would warrant a grade 3B designation; however, they were not assigned a grade in the current clinical context.

### Immunohistologic Features

**Fig 2:**
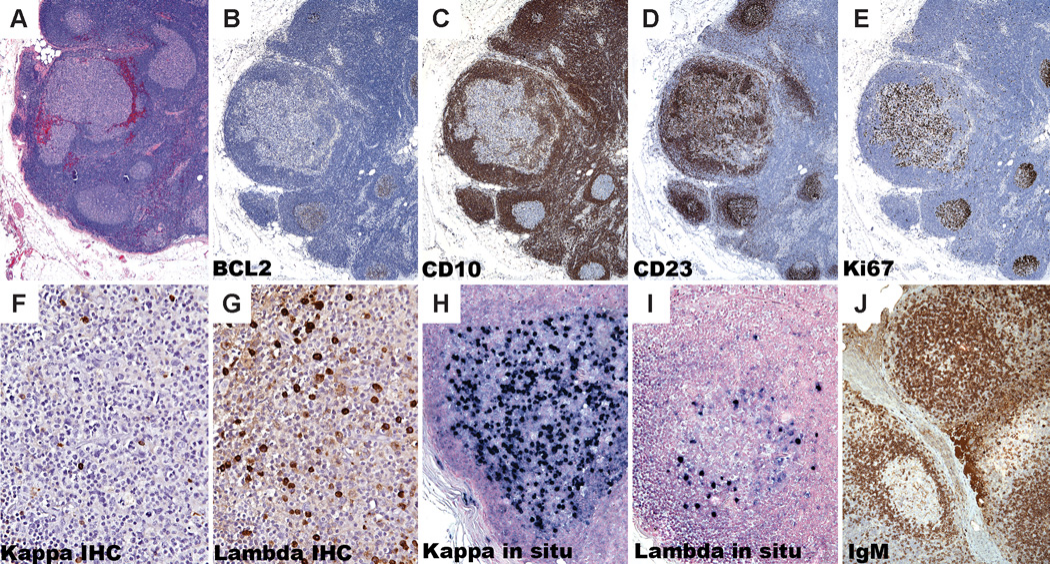
Immunohistochemistry of isolated atypical follicles. An axillary lymph node dissection in a 58 year-old woman with breast carcinoma (case 3) shows a lymph node with a cluster of follicles (A). BCL2 expression is absent in both the involved and uninvolved follicles (B) and the involved follicle shows diminished CD10 expression relative to the surrounding normal germinal centers (C). CD23 demonstrates an intact follicular dendritic network around the involved follicle (D). Ki-67 is polarized in surrounding reactive follicles, but is not polarized in the involved follicles (E). The involved follicle in case 1 shows lambda light chain-restricted B-cells (F and G). Case 2 shows highly atypical large cells that by situ hybridization (ISH) for immunoglobulin kappa and lambda light chains show kappa-specific RNA in the majority of the atypical cells, confirming light chain restriction in the involved follicle (H and I). A periaortic lymph node from a 53-year old woman (case 4) shows abnormal strong IgM protein expression in the centroblasts of an involved follicle whereas the uninvolved follicle shows a weak dendritic pattern of IgM reactivity, which is typically seen in normal follicles (J).

**Fig 3:**
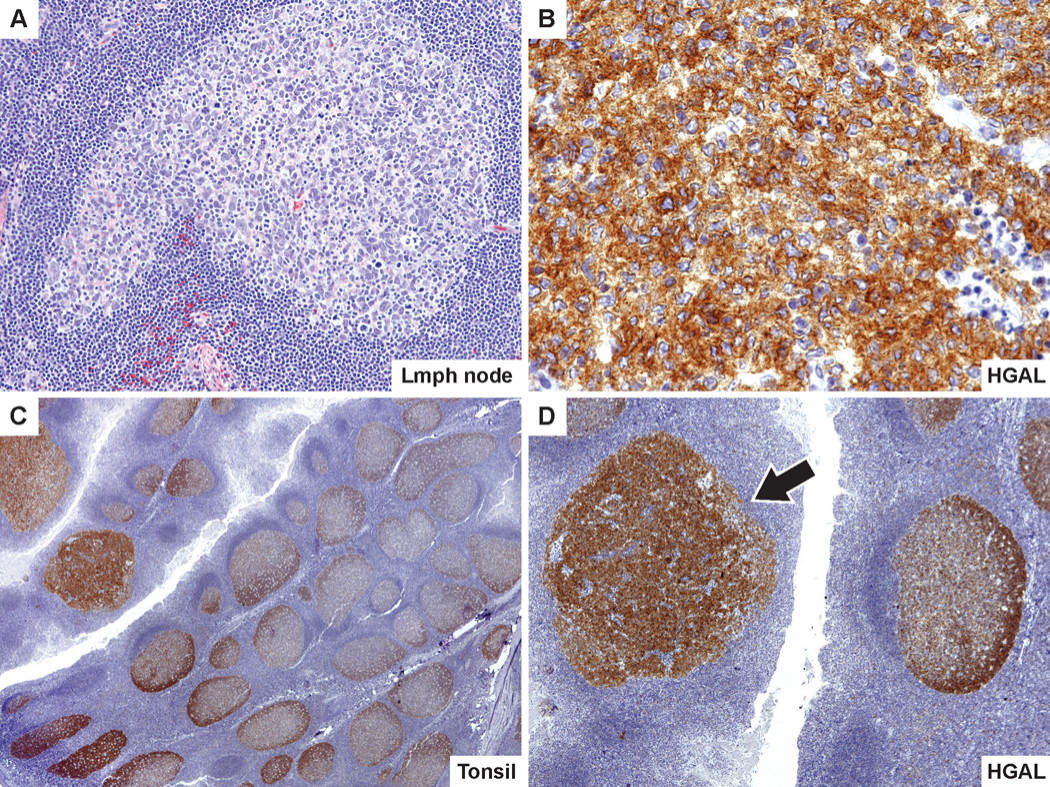
Atypical expression of HGAL in isolated atypical follicles. An axillary lymph node in a 53-year old woman (case 2) shows a single atypical follicle with pleomorphic large cells overexpressing HGAL (A and B). Sections of the tonsil in a 6-year old boy stained with HGAL, show preservation of overall architecture with numerous normal reactive follicles and a gradient of HGAL staining with higher intensity in the dark zone. In the atypical follicle (indicated by arrow in panel D), HGAL staining is abnormal and shows overexpression throughout the affected follicle (C and D).

The presence of a subset of markedly atypical follicles intermixed with normal reactive follicular hyperplasia led us to seek further immunohistochemical and molecular characterization of these follicles. The immunohistologic findings are summarized in Table 3 and representative images are illustrated in Figs 2 and 3. The germinal center origin of the large cell proliferations was demonstrated by positivity for one or more of the following germinal center B-cell markers, CD10 (6 of 7), BCL6 4 of 4) and HGAL (2 of 2). All seven cases lacked coexpression of BCL2 with a germinal center marker by immunohistochemistry, which is typical of the cases of follicular lymphoma *in situ* described thus far (Fig 2, panels A-B). CD10 showed normal expression in five cases; however, CD10 was absent or weak in the neoplastic follicles of cases 3 and 4, in comparison to the surrounding normal follicles in each case (Fig 2, panel C). The atypical centroblasts were present within well-organized follicular dendritic networks as demonstrated by CD21, CD23 or D2-40 immunostaining in five of the seven cases, supporting the follicular nature and excluding a small focus of diffuse large B cell lymphoma (Fig 2, panel D). The morphologic absence of polarization was probed with a Ki-67 stain, which demonstrated a strong but patchy staining pattern and yielded no subtle support for even poorly delineated light and dark zones (Fig 2, panel E).

**Table 3:** Summary of Immunohistologic Findings.

Case	CD20	CD3	CD10	BCL2	BCL6	HGAL	IGM	IRF4	Ki67	Dendritic markers CD21, CD23, D240	IG Light Chain Restriction
**1**	POS	NEG	Normal	NEG	ND	ND	POS	ND	ND	Dendritic meshwork present (CD21)	Lambda (IHC)
**2**	POS	NEG	Normal	NEG	POS	POS, strong	POS	NEG	50%, lack of polarization	Dendritic meshwork present (all three)	ND
**3**	POS	NEG	NEG	NEG	POS	ND	NEG	ND	50%, lack of polarization	Dendritic meshwork present (CD23)	Kappa (in situ)
**4**	POS	NEG	Weak	NEG	POS	ND	POS	ND	ND	ND	Non-contributory (IHC and in situ)
**5**	POS	NEG	Normal	NEG	POS	POS, strong	NEG	NEG	ND	Dendritic meshwork present (all three)	Lambda (IHC)
**6**	POS	NEG	Normal	NEG	ND	ND	NEG	ND	60%, lack of polarization	Dendritic meshwork present (CD21)	Non-contributory (IHC)
**7**	POS	Weak	Normal	NEG	ND	ND	ND	ND	ND	ND	ND

Table legend 3. Atypical large cells in involved follicles were scored POS (positive) or NEG (negative); ND: Not done due to insufficient tissue

A more direct assessment of clonality was pursued with kappa and lambda light chain staining. Although not informative in every case, three cases showed distinct staining patterns between the involved and uninvolved follicles. Cases 1 and 5 show lambda light chain restriction in the involved follicles and case 3 shows kappa light chain restriction (Fig 2, panels F–I). In each case, this provides further support for the clonal nature of the B-cells in the involved follicles.

An IgM immunostain was performed in six cases of which three showed an abnormal membranous pattern of staining (Fig 2, panel J). Whereas the surrounding unremarkable germinal centers show the expected weak dendritic pattern of IgM reactivity, the involved follicles show strong cytoplasmic staining, a feature associated with malignant follicles. One case (case 7) showed aberrant coexpression of CD3 in the atypical cells, a finding absent in the surrounding germinal centers.

Two cases (2 and 5) demonstrated altered expression of germinal center B-cell marker, HGAL, which show overexpression of the protein throughout the affected follicle. In comparison, the surrounding reactive follicles show a gradient of HGAL staining with higher intensity in dark zones, which matches the polarization of normal germinal centers (Fig 3).

### Fluorescence *in situ* hybridization and genotypic features

Six of seven cases with available material were tested by FISH for the presence of *BCL2* gene rearrangement. None were positive, which is in keeping with over 50 percent of grade 3 follicular lymphomas lacking the *IGH-BCL2* rearrangement. Two cases were further tested by FISH for *BCL6* and *IRF4* gene rearrangements (case 2, lymph node; case 5, tonsil) and were found to be negative (Table 4).

**Table 4:** Summary of FISH and Molecular Findings.

Case	BCL2 FISH	BCL6 FISH	IRF4 FISH	Molecular Findings
1	NEG	ND	ND	Polyclonal; not microdissected due to lack of tissue
2	NEG	NEG	NEG	Clonal population detected in microdissected sample
3	NEG	ND	ND	Polyclonal, although apparent paucity of B-cells in microdissected sample
4	NEG	ND	ND	ND
5	NEG	NEG	NEG	Polyclonal in microdissected sample
6	NEG	ND	ND	Polyclonal; not microdissected due to lack of tissue
7	ND	ND	ND	ND

Table 4 legend. Atypical large cells in involved follicles were scored POS (positive) or NEG (negative); ND: Not done due to insufficient tissue

Molecular PCR studies for *IGH* gene rearrangements were pursued in five cases, all of which initially failed to show a B-cell receptor gene rearrangement. In three cases with available material molecular studies were pursued on microdissected samples. Among the three cases analyzed by microdissection, two cases had only one follicle each that was involved. The third case had two closely apposed follicles, which were pooled. Two of these cases were successfully amplified, and a clonal amplification was further detected on a microdissected sample of case 2 among the B-cells comprising the involved follicles but not in the uninvolved lymph node follicles from the same case (Table 4).

## Discussion

Isolated follicles enriched for centroblasts in otherwise reactive lymph nodes pose a distinct diagnostic challenge. These cases are difficult to classify and in the current diagnostic framework, the differential diagnoses would include a focus of diffuse large B cell lymphoma, partial involvement by grade 3 follicular lymphoma, follicular lymphoma *in situ*, or an unusual reactive phenomenon. The seven cases we describe are distinctly different from previously reported cases of follicular lymphoma *in situ* in that they demonstrate a predominance of centroblasts and lack expression of BCL2 protein.

The cases presented herein, despite the absence of the *IGH-BCL2* translocation and the corresponding BCL2 protein overexpression, demonstrate aberrant morphologic and immunophenotypic features that are typical of lymphoma. These cases are characterized by pleomorphic cytology with irregular nuclear contours, large nuclear size and atypical mitotic figures. These cells are arranged in clusters amid intact follicular dendritic networks and lack key features of the normal germinal center, including tingible body macrophages and polarization. Furthermore, these cases demonstrate altered expression of germinal center markers CD10 and HGAL, co-expression of CD3, abnormal proliferation indices and disturbed IgM staining patterns. Each of these features can be seen in the setting of lymphoma, but is absent in the physiologic germinal center. Clonality studies, including light chain expression analysis and molecular assessment of the *IGH@* gene rearrangement provide further support for the clonal nature of these follicles in the majority of the cases. Three cases had kappa or lambda light chain restricted B-cells and a fourth case was clonal by gene rearrangement studies following microdissection. The caveat that clonality studies in limited samples may demonstrate spurious clones when only a few B-cells are present should be kept in mind. Moreover, even non-neoplastic conditions can demonstrate clonal populations in the context of normal reactive germinal centers and hyperplastic marginal zones. [25–26] Therefore, our diagnoses are based predominantly on the altered morphology and immunoarchitecture, coupled with the markedly atypical cytologic findings, in the involved follicles. Immunohistochemistry shows decreased to absent CD10 expression in two cases, increased HGAL reactivity compared to background follicles in two cases, and light chain-restricted cells confined to the aberrant follicles in three cases of which one was also confirmed by microdissection of abnormal follicles with detection of clonal amplification and suggests that these findings represent a distinct pathologic process.

Although there is some support for neoplasia, the appropriate classification is unclear in the currently used system. As the proliferations are found only within follicular dendritic networks, diffuse large B cell lymphoma was excluded. The germinal center origin of the cells was supported by expression of CD10, BCL6, and/or HGAL, helping exclude lymphomas of non-germinal center origin. The atypical follicles were single or scattered amongst the germinal centers of a reactive pattern lymph node, which would be atypical for a definitive diagnosis of follicular lymphoma demonstrating partial involvement. To further exclude partial involvement by follicular lymphoma, and as recommended by Carbone and Santoro,[23] each patient received a complete staging workup to exclude additional sites of lymphadenopathy and associated involvement by indolent or aggressive lymphoma. In a subset of patients this included a bone marrow biopsy. None of the patients had evidence of systemic lymphoma at time of diagnosis or at available follow-up (up to 44 months later). Despite the concerning morphologic features that raise consideration of an aggressive lymphoma, such as diffuse large B cell lymphoma, not otherwise specified, the presence of these scattered atypical follicles did not require aggressive clinical management. Our previous studies on the germinal center B-cell associated protein, HGAL, showed that increased expression of HGAL is correlated with low stage disease and decreased capacity for dissemination.[27–28] None of the cases with increased HGAL expression were associated with systemic lymphoma, supporting this finding.

The t(14;18)(q32;q21) translocation involving the *IgH* and *BCL2* genes that results in the overexpression of the anti-apoptotic protein BCL2, is the initiating event and genetic hallmark of FL.[29] Up to 10% of FL lack the t(14;18) translocation and the vast majority of these cases also lack expression of the BCL2 protein. Lack of BCL2 expression is particularly enriched in grade 3 FL, where only 50–70% of cases express the BCL2 protein in neoplastic B-cells within the follicle.(24) Recent investigations have shown that t(14;18)-negative FL is characterized by distinct microRNA profiles, an increased proliferative capacity and a late-germinal center immunophenotype, while t(14;18)-positive FL that lack BCL2 retain the full germinal center phenotype similar to t(14;18)-positive, BCL2-positive FL.[30]

Jegalian and colleagues also reported *t(14;18)/IGH-BCL2* translocation-positive cases that lacked BCL2 reactivity in cases of FL associated with FLIS.[1] In addition, a single case report documented an additional point mutation within the *BCL2* breakpoint region in the FL component of a case with simultaneous FL and FLIS.[31]These findings suggest a process of clonal evolution in cases with FLIS associated with FL. Recent gene array comparative genomic hybridization studies have demonstrated a continuum of cytogenetic findings ranging from none in reactive follicular hyperplasia, few in FLIS and more numerous chromosomal aberrations in fully developed follicular lymphoma.[32–33] These findings support a model of increasing cytogenetic complexity with progression of follicular lesions. Such studies would be interesting in cases similar to the ones we describe to ascertain if the miRNA and cytogenetic profiles more closely resemble those of FLIS, partial involvement by FL or fully developed follicular lymphoma.

FLIS is recognized as a follicular proliferation of monoclonal BCL2+ CD10+ lymphoid cells within scattered germinal centers of a lymph node with otherwise intact architecture. Cases described to date have been comprised of predominantly centrocytes and contained the *IGH-BCL2* translocation. The seven cases we describe are distinctly different from the previously reported cases in that they demonstrate a predominance of centroblasts and lack expression of BCL2.

While the clinical significance of FLIS will depend largely on the results of long-term follow-up, initial studies suggest progression to follicular lymphoma is an infrequent event.[34] It may be many years before the true relationship to systemic lymphoma is uncovered given that even low-grade FL tends to be an indolent disease that many oncologists manage with minimal therapy such as rituximab or a ‘watch-and-wait’ approach.[35–37] Conceivably, decades of follow-up may be necessary in prospective trials to determine if there is an increased risk for the development of lymphoma in these patients, and, even then, early diagnosis in this setting may not alter clinical management. The relationship between the current cases and follicular lymphoma in situ is unclear and whether these could represent a variant of follicular lymphoma in situ with grade 3A or 3B morphology should be evaluated in future investigations.

Early detection of an aggressive B-cell lymphoma or carcinoma may lead to immediate intervention. Our cases showed involvement of rare scattered follicles in an architecturally intact lymph node, but contained increased centroblasts histologically consistent with grade 3B FL. Just as in typical FLIS, these cases were incidental findings in lymph nodes removed for various reasons and the patients did not show clinical or radiological evidence of lymphoma at the time of presentation. It is critical to recognize such cases, both for further study, and to potentially avoid overly aggressive treatment of an indolent lesion.
